# Inappropriate use of the title 'chiropractor' and term 'chiropractic manipulation' in the peer-reviewed biomedical literature

**DOI:** 10.1186/1746-1340-14-16

**Published:** 2006-08-22

**Authors:** Adrian B Wenban

**Affiliations:** 1 Collaborator, Unidad de Investigación en Servicios Sanitarios, Institut Municipal d'Investigació Mèdica (IMIM), Barcelona, Spain

## Abstract

**Background:**

The misuse of the title 'chiropractor' and term 'chiropractic manipulation', in relation to injury associated with cervical spine manipulation, have previously been reported in the peer-reviewed literature.

The objectives of this study were to -

**1) **Prospectively monitor the peer-reviewed literature for papers reporting an association between chiropractic, or chiropractic manipulation, and injury;

**2) **Contact lead authors of papers that report such an association in order to determine the basis upon which the title 'chiropractor' and/or term 'chiropractic manipulation' was used;

**3) **Document the outcome of submission of letters to the editors of journals wherein the title 'chiropractor', and/or term 'chiropractic manipulation', had been misused and resulted in the over-reporting of chiropractic induced injury.

**Methods:**

One electronic database (PubMed) was monitored prospectively, via monthly PubMed searches, during a 12 month period (June 2003 to May 2004). Once relevant papers were located, they were reviewed. If the qualifications and/or profession of the care provider/s were not apparent, an attempt was made to confirm them via direct e-mail communication with the principal researcher of each respective paper. A letter was then sent to the editor of each involved journal.

**Results:**

A total of twenty four different cases, spread across six separate publications, were located via the monthly PubMed searches. All twenty four cases took place in one of two European countries.

The six publications consisted of four case reports, each containing one patient, one case series, involving twenty relevant cases, and a secondary report that pertained to one of the four case reports. In each of the six publications the authors suggest the care provider was a chiropractor and that each patient received chiropractic manipulation of the cervical spine prior to developing symptoms suggestive of traumatic injury.

In two of the four case reports contact with the principal researcher revealed that the care provider was not a chiropractor, as defined by the World Federation of Chiropractic. The authors of the other two case reports did not respond to my communications. In the case series, which involved twenty relevant cases, the principal researcher conceded that the term chiropractor had been inappropriately used and that his case series did not relate to chiropractors who had undergone appropriate formal training. The author of the secondary report, a British Medical Journal editor, conceded that he had misused the title chiropractor. Letters to editors were accepted and published by all four journals to which they were sent. To date one of the four journals has published a correction.

**Conclusion:**

The results of this year-long prospective review suggests that the words 'chiropractor' and 'chiropractic manipulation' are often used inappropriately by European biomedical researchers when reporting apparent associations between cervical spine manipulation and symptoms suggestive of traumatic injury. Furthermore, in those cases reported here, the spurious use of terminology seems to have passed through the peer-review process without correction. Additionally, these findings provide further preliminary evidence, beyond that already provided by Terrett, that the inappropriate use of the title 'chiropractor' and term 'chiropractic manipulation' may be a significant source of over-reporting of the link between the care provided by chiropractors and injury. Finally, editors of peer-reviewed journals were amenable to publishing 'letters to editors', and to a lesser extent 'corrections', when authors had inappropriately used the title 'chiropractor' and/or term 'chiropractic manipulation'.

## Background

Figures quoted in the peer-reviewed literature for the incidence of cervical manipulative therapy associated vertebrobasilar accidents (VBA), based largely on various types of retrospective surveys, range from a high of 1:20,000 cervical manipulations [1] to a low of 1:5.85 million cervical manipulations [2]. Due to the rarity of these injuries and the methodological weaknesses that affected all relevant research carried out to date, the true incidence of VBA and other injuries due to spinal manipulative therapy (SMT) provided by qualified chiropractors remains unknown. Furthermore, sources of over-reporting and under-reporting of such injuries remain poorly explored. A number of authors have argued adamantly, also based largely on poorly controlled retrospective surveys, that complications arising from the use of SMT by chiropractors are likely to be far more frequent than reported in the peer-reviewed literature due to what they believe is an extraordinarily high level of under-reporting. An example is Professor Edzard Ernst, Director of Complementary Medicine, Peninsula Medical School at the Universities of Exeter and Plymouth, who recently stated;

'One gets the impression that the risks of spinal manipulation are being played down, particularly by chiropractors. Perhaps the best indication that this is true are estimates of incidence rates based on assumptions, which are unproven at best and unrealistic at worse. One such assumption, for instance, is that 10% of actual complications will be reported. Our recent survey, however, demonstrated an underreporting rate of 100%. This extreme level of underreporting obviously renders estimates nonsensical'. [3]

Less discussed, and less researched, are potential sources of over-reporting of chiropractic SMT related complications. A number of authors have pointed out that the presence of injury subsequent to SMT does not necessarily indicate a cause and effect relationship, but merely indicates a temporal relationship. In such cases an already existing and evolving pathology (artery dissection or disc lesion) may have in fact been the cause of the symptoms that prompted the patient to seek the practitioner for relief. [4] Leboeuf-Yde and co-authors [5], by way of a case series, described a number of examples of severe or fatal symptoms that might have been attributed to SMT provided by chiropractors, had the temporal relationship between the evolution of each patients respective condition and the chiropractic care that they were scheduled to receive been different. The authors concluded;

'The incidence of iatrogenic events that occur after chiropractic treatment is unknown. Such events are generally thought to be under-reported. However, the possibility of over-reporting should also be kept in mind'.

Another source of over-reporting arises out of identifying a care provider, whose application of SMT is associated with injury, as a chiropractor when the care provider is not in fact a qualified chiropractor. Terrett [6] carried out a review with the objective of determining how the words chiropractic and chiropractor had been used in publications reporting complications from cervical SMT. After reviewing the literature, and contacting many of the authors involved, Terrett [6] concluded;

'The words chiropractic and chiropractor have been incorrectly used in numerous publications dealing with SMT injury by medical authors, respected medical journals and medical organizations..... Such reporting adversely affects the reader's opinion of chiropractic and chiropractors'.

The objectives of the present study were to;

**1) **Prospectively monitor one electronic database (PubMed) across the course of 1 year (June 2003 – May 2004) for papers reporting an association between chiropractic, or chiropractic manipulation, and any form of injury.

**2) **Contact lead authors of those papers that report such an association in order to determine the basis upon which the title 'chiropractor' and/or term 'chiropractic manipulation' were used;

**3) **Document the outcome of submission of letters to the editors of journals in which the title 'chiropractor' and term 'chiropractic manipulation' had been misused and resulted in the over-reporting of chiropractic induced injury.

## Methods

The peer-reviewed biomedical literature was monitored prospectively, via monthly searches of one electronic database (PubMed), during a 12 month period (June 2003 – May 2004). The monthly PubMed searches used the key terms, 'chiropractic, manipulation, complication'. Once relevant articles were located they were reviewed with a view to determine the qualifications, educational background and professional identity of the care provider. If the paper failed to provide such details, efforts were made to contact the lead author of the article via e-mail in an attempt to clarify whether the care provider was in fact a qualified chiropractor. If the personal response from the paper's lead author confirmed the inappropriate use of the title or term, a letter outlining the case was written and submitted to the involved journal editor with the request that a correction be published.

For the purpose of this review the term 'qualified chiropractor' was defined in accordance with World Federation of chiropractic (WFC) policy statements [7] as quoted in WFC Secretary General David Chapman-Smith's book, *'The Chiropractic Profession. Its Education, Practice, Research and Future Directions' *[8], wherein it is states;

'The World Health Organization (WHO) has granted official status to the World Federation of Chiropractic (WFC) as an affiliated nongovernmental organization (NGO). In its policy statement on the "Use of the title Chiropractor," the WFC states that chiropractors must be;

"...graduates of chiropractic educational programmes that are formally accredited by a chiropractic accreditation agency or an alternative government-recognized accreditation process in the country in question, or that are recognized and approved on an interim basis within the terms of the World Federation of Chiropractic's Tokyo Charter by the national association of chiropractors in the country in question".

This position paper was revised in 2003 to extend the range of the policy to cover the term 'chiropractic' as well'.

## Results

A total of twenty four separate cases were located via the PubMed searches (See Table 1.) and were included in this review. These twenty four cases were spread across six separate publications [9-14]. The six publications were;

**Table 1:** Summary of the findings of this prospective review – wherein reports inappropriately linked the title chiropractor, and/or term chiropractic manipulation, to injury (n = 24).

Author* Ref no.	**Country of origin**	**No. of subjects**	**Journal**	**Study type**	**Injury**	**Care provider**
Beck^9^	Germany	1	JNNP†	Case report	DT‡	Heilpraktiker
Saxler^10^	Germany	1	ZOIG§	Case report	SEH||	Unknown
Oheler^11^	Germany	1	Orthop¶	Case report	VAD** (Bilateral)	Unknown
Menendez^12^	Spain	1	Rev Neurol††	Case report	VAD**	Unqualified manipulator
Dziewas^13^	Germany	20	Journal of Neurology	Case series	ICAD‡‡(5) VAD** (14) ICAD+VAD(1)	All non-chiropractors
Markovitch^14^	Germany	1	Br Med Journal	2° report of Beck^9^	DT‡	Heilpraktiker

* Only first author included

† Journal of Neurology and Neurosurgical Psychiatry

‡ Dural Tear

§ Zietschrift fur Orthopadie und Ihre Grenzgebiete

|| Spinal Epidural Hematoma

¶ Orthopade

** Vertebral Artery Dissection

†† Revista de Neurologia

‡‡ Internal Carotid Artery Dissection

• Four separate case reports (three from Germany [9-11], and one from Spain [12]),

• One case series [13], containing twenty relevant cases (from Germany), and

• A secondary report, published in an English medical journal [14], which briefly summarised the findings from one of the above mentioned case reports [9].

In relation to each of the six publications attempts were made to contact the principal author in order to find out more information about the qualifications of the practitioners involved. Prior to contacting the principal researchers the president of the WFC-affiliated national chiropractic association in that country was contacted in order to ask for permission to proceed with my research-related communication. This was done with the hope of complying with the WFC's policy statement titled 'Communication Protocols on Education and Research' [15], which states;

'Should an educational institution (*or individual*) from one country wish to establish a chiropractic research initiative in another country, notification shall be given to any existing national association of chiropractors within that other country...'

In each of the 6 publications the authors identified the care provider as a 'chiropractor' and stated that each patient received 'chiropractic manipulation' of the cervical spine prior to developing symptoms suggestive of traumatic injury.

In two [9,12], of the four case reports contact with the principal researcher revealed that the care provider was not a chiropractor and that the terms 'chiropractic manipulation' and 'chiropractor' were inappropriately used. The authors of the other two case reports [10,11], did not respond to my e-mail communications. Regarding the case series involving twenty relevant cases [13], the principal researcher initially related that he did not know whether the care providers in each case were, or were not, qualified chiropractors. In a subsequent letter to the editor of the Journal of Neurology [16] the lead author of that case series conceded that none of the care providers in his case series were qualified chiropractors (See Table 1.).

All four journals, to which letters were sent (British Medical Journal, Journal of Neurological and Neurosurgical Psychiatry, Journal of Neurology, Revista de Neurología), accepted the letter to editor for publication [17-20]. To date only one journal, the British Medical Journal, has published a correction [21].

## Discussion

As already mentioned, Terrett [6] has carried out a study with the objective of determining how the words chiropractic and chiropractor have been used in publications reporting complications from cervical SMT. Furthermore, he pointed out that in countries without chiropractic registration it can be difficult to determine the educational and professional background of the provider of care that has been associated with harm. Terrett revealed that errors regarding terminology and professional identity have been documented, in one or more cases, in India, Ireland, Italy, Taiwan, France, and Germany. The results of the present study suggest that errors regarding terminology and professional identity are still taking place in Germany [9,13], and that Spain [12] and England [14] can now also be added to that list.

Terrett's review [6] revealed that many cases of complication after manipulation described in the medical literature as "chiropractic complications" are found to be, on closer inspection, either

(a) medical misrepresentation of the literature;

(b) inaccurate reporting by medical authors; or

(c) inaccurate reporting by medico-legal journalists.

Reflecting upon the present study in light of Terrett's classification, we find that two case studies [9,12], and one case series [13] are examples of inaccurate reporting by medical authors and that the secondary report, published in an English medical journal [14], that briefly summarised the findings from one of the above mentioned case reports [9], was in part the result of inaccurate reporting by the involved BMJ editor, and is therefore an example of medical misrepresentation of the literature.

It is important for research attempting to document associations between the care provided by chiropractors and harm, and/or benefits, to realize that chiropractic is a profession and not just a procedure. That such is the case is reflected in WHO Guidelines on Basic Training and Safety in Chiropractic [22];

'***Chiropractic ***– A health profession concerned with the diagnosis, treatment and prevention of disorders of the musculoskeletal system, and the effects of these disorders on the nervous system and general health.'

Furthermore, membership in the chiropractic profession is predicated on detailed globally agreed upon educational standards. The European Council on Chiropractic Education (ECCE) is an international autonomous organization established by the chiropractic profession in Europe to accredit and re-accredit institutions providing undergraduate chiropractic education and training. The principal goal of the ECCE is to assure the quality of chiropractic undergraduate education and training against a set of educational Standards. It is the only chiropractic educational accreditation agency serving Europe that is a member of the Council on Chiropractic Education International (CCEI), and that is recognized by the profession and the other Councils on Chiropractic Education around the world. Through its membership with CCEI, the ECCE adheres to the CCEI Model Standards that include educational criteria and accreditation procedures. Such adherence indicates essential equivalence of the educational standards of all CCEI member agencies and, thereby, the competency of individuals graduating from institutions accredited by ECCE and all member Councils on Chiropractic Education [23].

ECCE Standards specify that the duration of a chiropractic programme last for at least five full-time academic years and are based on the following definition and description of a chiropractor:

'The chiropractor is concerned with the health needs of the public as a member of the healing arts. He/she gives particular attention to the relationship of the structural and neurological aspects of the body in health and disease. He/she is educated in the basic and clinical sciences as well as in related health subjects. The purpose of his/her professional education is to prepare for practice as a primary care provider. As a portal of entry to the healthcare system, the chiropractor must be well educated to diagnose, to care for the human body in health and disease, and to consult with, or refer to, other healthcare providers'. [23]

To describe someone as a chiropractor simply because that person performs SMT, but without knowing that person's educational background or professional affiliations, is clearly inappropriate. However, that seems to be the basis upon which the authors of the six articles of this review proceeded.

Chiropractors who feel an injustice has been committed when case reports inappropriately link their profession with some form of harm might consider the following. The scientific process is not over with the publication of an article; it's up to the scientist's or clinician's peers to review works in journals and critique them with letters to the editor. If a publication's errors go unchallenged, they are the fault of the reader as much as the person who submitted the paper for publication.

The researchers who have published these cases probably did not have malicious intentions towards chiropractic. I believe that case studies of 'manipulation induced injury' readily catch the eye of readers and journal editors alike so that such papers make for an easy publication. Furthermore, I get the impression, from my communications with the principal researchers involved, that they are using the title 'chiropractor' as if it is synonymous with 'spinal manipulator' and the term 'chiropractic manipulation' as if it is synonymous with 'spinal manipulation'.

Unfortunately, the case series by Dziewas et al. [13], which incorrectly suggested that twenty cases of carotid artery dissection (CAD) were caused by qualified chiropractors has been quoted by at least four subsequent publications [24-27]. This is another form of what Terrett [6] termed "inaccurate reporting", wherein the original publication, by Dziewas et al. [13], does attribute the injuries to chiropractors, but personal communication with the author, and subsequent letters to the editor [16,20], revealed a different scenario. This form of inaccurate reporting likely adds significantly to the over-reporting of chiropractic-related injury. In support of the authors of those four subsequent publications it should be noted that all three of the reviews [24-26], and the one commentary [27] were submitted for publication subsequent to the date when my letter to the editor of the Journal of Neurology [20], and the reply by Dziewas [16], made public the fact that the care providers of the 20 relevant cases they reported on were not qualified chiropractors.

It is noted with dismay that despite all the discussion, via letters to editors, in relation to the inappropriate use of the title chiropractor and term chiropractic manipulation, that a more recently published retrospective survey from Germany [28] used the title, *Vertebral artery dissections after chiropractic neck manipulation*, despite the fact that only 4/36 (11%) of the cases reportedly involved a care provider identified as a chiropractor. The largest group of providers, 18/36 (50%), linked to injury through their use of SMT, were orthopaedic surgeons. Surely if the title of that paper was going to be associated with one particular profession, or specialty, it should have been orthopaedic surgeons rather than chiropractors. Furthermore, given the propensity for publications from Germany to inappropriately use the title 'chiropractor', it may yet prove to be the case that none of the four chiropractic-related injuries identified by that retrospective review were the result of care provided by qualified chiropractors.

This prospective review suffers from a number of limitations;

• Only one electronic data base (PubMed) was monitored across the 12 month. Had more data bases been included more relevant studies may have been detected,

• The search strategy used for this review was very basic and included only three search terms. A more detailed search strategy and the inclusion of more search terms may have resulted in the detection of more relevant studies,

• The means by which authors of relevant papers were contacted was limited to e-mail. Authors of two of the six relevant papers detected via this review's search strategy did not respond to this author's e-mails. The professional identity of the care providers in those two case studies therefore remains unknown. A more thorough attempt to communicate with authors of relevant papers, beyond e-mail, may have been more successful in eliciting responses.

The true incidence of this form of over-reporting, wherein a care provider, whose application of SMT is associated with an injury, is inappropriately identified as a chiropractor, has not been documented. However, based on the preliminary findings provided by Terrett's previous work [6] and the present prospective review, further investigation, aimed towards determining the extent to which the inappropriate use of terminology contributes to the over-reporting of chiropractic induced injury, seems warranted. Further research in this area might also explore whether the title 'chiropractor' and term 'chiropractic manipulation' are being misused only in relation to harm caused by non-chiropractors, or if the same misuse is taking place in relation to descriptive studies documenting benefits. That is to say it might also be informative, in determining whether any bias exists, to look at whether the term and title are also being inappropriately used in studies documenting some patient benefit due to this type of care when provided by non-chiropractors.

Moreover it is recommended that authors of future publications, aimed at exploring the link between SMT and injury, should very clearly define the terminology used and clearly delineate the training and professional identification of those care providers whose care is subsequently deemed to be in some way associated with injury.

## Conclusion

The results of this study suggest that the words 'chiropractor' and 'chiropractic manipulation' are often used inappropriately by European biomedical researchers in relation to the reporting of injuries associated with cervical spine manipulation. Furthermore, the findings of this year-long prospective review provide further preliminary evidence, beyond that already provided by Terrett [6], that the inappropriate use of the title 'chiropractor' and term 'chiropractic manipulation' may be a significant source of over-reporting of the link between chiropractic care and injury. Editors of peer-reviewed scientific journals were amenable to publishing 'letters to editors', and to a lesser extent 'corrections', pertaining to original research that had inappropriately used the title 'chiropractor' and/or term 'chiropractic manipulation'.

## Competing interests

The author declares that he is a practicing chiropractor (part-time), an Associate Governor of the Australian Spinal Research Foundation, a doctoral student, and a European Chiropractic Union nominated, and ECCE elected, member of the ECCE.

No external funds or grants were used for this study.

## Authors' contributions

The author designed the study, wrote the proposal, reviewed papers, wrote to presidents of WFC affiliated national chiropractic associations, wrote to the authors of relevant papers, wrote letters to editors and wrote this paper.
